# Supplementary material for: Identification of photoperiod-induced specific miRNAs in the adrenal glands of Sunite sheep (Ovis aries)
Source: Front Vet Sci. 2022 Jul 22;9:888207. doi: 10.3389/fvets.2022.888207 (PMC9354845; doi:10.3389/fvets.2022.888207)
Supplement: Supplementary file 1 [file Data_Sheet_1.ZIP › Supplementary file/miRDeep2 file.zip!/miRDeep2 file/Know_Structure/LP42A3/oar-mir-143.pdf]

miRBase precursor	: oar-mir-143
Total read count	: 254290
oar-miR-143 read count	: 252363
remaining reads	: 1927

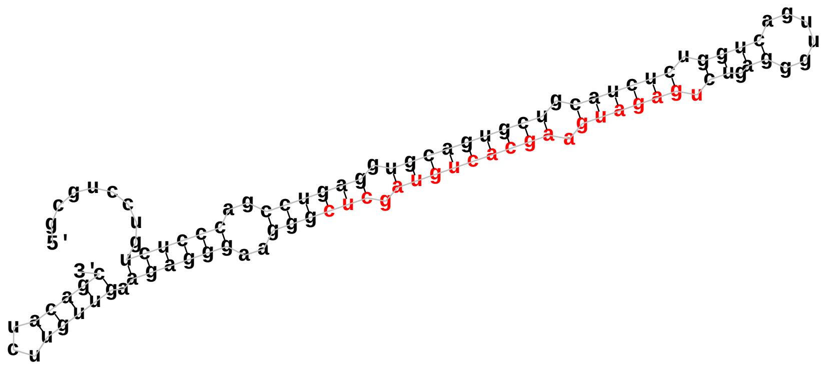

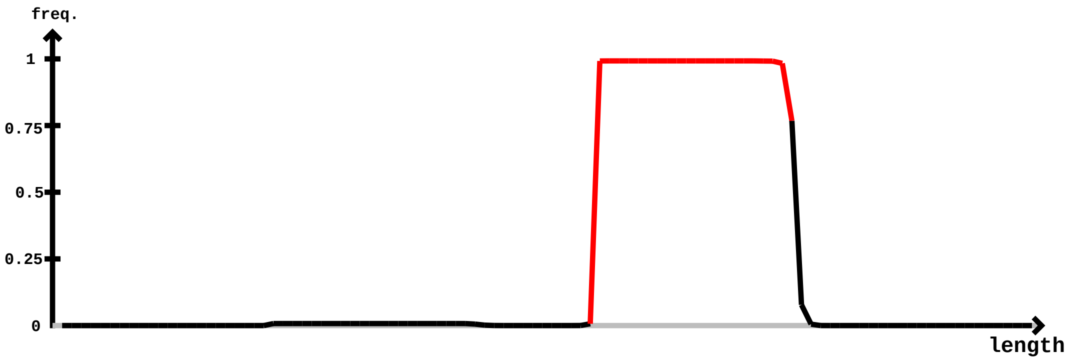

oar-miR-143

```
5'- gcguccugucucccagccugaggugcagugcugcaucucuggucaguugggagucugagaugaagcacuguagcucgggaagggagaaguuguucuacagc  -3'    exp
    ........((((((..((((((.(((((((((.((((((.((((......)).)).)))))).))))))))).))))))..)))))).(((((...)))))   reads       mm          sample
    ....................aggugcagugcugcaucucugg...........................................................   2           0           seq
    ....................aggugcagugcugcaucucuggu..........................................................   2           0           seq
    .....................ggugcagugcugcaucucug............................................................   14          0           seq
    .....................ggugcagugcugcaucucugg...........................................................   507         0           seq
    .....................ggugcagugcugcaucucuggu..........................................................   995         0           seq
    .....................ggugcagugcugcaucucugguc.........................................................   350         0           seq
    .....................ggugcagugcugcaucucuggucа........................................................   48          0           seq
    .....................ggugcagugcugcaucucuggucaguu.....................................................   1           0           seq
    ......................gugcagugcugcaucucuggu..........................................................   1           0           seq
    ......................gugcagugcugcaucucugguc.........................................................   1           0           seq
    .........................................gucaguugggagucugagaugaagcacuguagcuc.........................   1           0           seq
    ...............................................ugggagucugagaugaagcacug...............................   1           0           seq
    ...................................................agucugagaugaagcacuguagcu..........................   2           0           seq
    ...................................................agucugagaugaagcacuguagcucg........................   1           0           seq
    ....................................................gucugagaugaagcacuguagc...........................   1           0           seq
    .....................................................ucugagaugaagcacuguagc...........................   3           0           seq
    .....................................................ucugagaugaagcacuguagcu..........................   2           0           seq
    .....................................................ucugagaugaagcacuguagcuc.........................   49          0           seq
    ......................................................cugagaugaagcacugu..............................   2           0           seq
    ......................................................cugagaugaagcacugG..............................   1           1           seq
    ......................................................cugagaugaagcacuguag............................   2           0           seq
    ......................................................cugagaugaagcacuguagc...........................   25          0           seq
    ......................................................cugagaugaagcacuguagcu..........................   373         0           seq
    ......................................................cugagaugaagcacuguagcuc.........................   1224        0           seq
    ......................................................cugagaugaagcacuguagcucg........................   51          0           seq
    ......................................................cugagaugaagcacuguagcucgg.......................   1           0           seq
    .......................................................ugagaugaagcacugua.............................   123         0           seq
    .......................................................ugagaugaagcacuguG.............................   1           1           seq
    .......................................................ugagaugaagUacugua.............................   5           1           seq
    .......................................................ugagaugaagcacuCua.............................   1           1           seq
    .......................................................ugagaugaagcacuguU.............................   1           1           seq
    .......................................................ugagaugaagcacuguag............................   109         0           seq
    .......................................................ugagauAaagcacuguag............................   1           1           seq
    .......................................................ugagauUaagcacuguag............................   1           1           seq
    .......................................................ugagaugaagcacuguagc...........................   2076        0           seq
```

oar-miR-143

```
gcguccugucucccagccugaggugcagugcugcaucucuggucaguugggagucugagaugaagcacuguagcucgggaagggagaaguuguucuacagc
.......................................................ugagaugaagcacuguagcu..........................    54392    0    seq
.......................................................ugagaugaagcacuguagcuc.........................    174103   0    seq
.......................................................ugagaugaagcacuguagcucg........................    18430    0    seq
.......................................................ugagaugaagcacuguagcucgg.......................    1291     0    seq
.......................................................ugagaugaagcacuguagcucggg......................    10       0    seq
........................................................gagaugaagcacuguagcu..........................    19       0    seq
........................................................gagaugaagcacuguagcuc.........................    38       0    seq
........................................................gagaugaagcacuguagcucg........................    5        0    seq
.........................................................agaugaagcacuguagcu..........................    1        0    seq
.........................................................agaugaagcacuguagcucgg.......................    8        0    seq
..........................................................gaugaagcacuguagcucgg.......................    7        0    seq
..........................................................gaugaagcacuguagcucggg......................    4        0    seq
..........................................................gaugaagcacuguagcucggga.....................    2        0    seq
...........................................................augaagcacuguagcuc.........................    1        0    seq
...........................................................augaagcacuguagcucggga.....................    1        0    seq
```
